# Ultrasound-guided Hydrostatic Reduction of Acute Intussusception in Children at a Tertiary Care Center: An Observational Study

**DOI:** 10.31729/jnma.8878

**Published:** 2025-02-28

**Authors:** Ajay Kumar Yadav, Neha Yadav, Binit Dev, Sonia Dahal, Rohit Prasad Yadav, Sushil Taparia, Hiramani Patahak

**Affiliations:** 1 Department of Radiodiagnosis and Interventional Radiology, Birat Medical College & Teaching Hospital, Biratnagar, Morang, Nepal; 2 Department of Microbiology & Infectious Disease, Koshi Zonal Hopsital, Biratnagar, Morang, Nepal; 3 Department of Pediatrics, Birat Medical College & Teaching Hospital, Biratnagar, Morang, Nepal; 4 Department of General Surgery, Birat Medical College & Teaching Hospital, Biratnagar, Morang, Nepal; 5 Department of Pediatrics Surgery, Birat Medical College & Teaching Hospital, Biratnagar, Morang, Nepal

**Keywords:** *hydrostatic*, *intussusception*, *reduction*, *ultrasonography*

## Abstract

**Introduction:**

Intussusception is common cause of acute emergency responsible for bowel obstruction in infants and toddlers with peak age of incidence between 6-9 months. Ultrasound is imaging modality of choice for diagnosis of intussusception and ultrasound guided hydrostatic enema reduction is standard, nonsurgical, internationally preferred treatment modality for uncomplicated pediatric intussusceptions. This study was aimed to find the outcome of the procedure.

**Methods:**

An observational cross-section was carried in a tertiary care center in children presenting with intussusception. Ultrasoud guided hydrostatic reduction was done in all radiologically confirmed intussusception in children presenting to our tertiary hospital from February 2023 to July 2024 fulfilling the inclusion criteria after proper resuscitation. Patients having marked abdominal distension with features of peritonitis were excluded from the study. Ethical approval was obtained from Institutional Review Committee (Reference number: 287/2023).

**Results:**

Total 81 patients with 84 intussusceptions underwent ultrasound guided hydrostatic reduction with male to female ratio of 2.86 and mean age 9.49±8.43 months. Hydrostatic reduction was successful in 78 (92.85%) cases. Among successful reductions, 3 (3.84%) patients had recurrence and repeat successful hydrostatic reduction was done. Presenting complain seen were abdominal pain 81 (100%) , vomiting 72 (88.89%). Ileocolic intussusception was seen in 74 (88.09%).

**Conclusions:**

The success rate of ultrasound-guided hydrostatic reduction using normal saline for uncomplicated intussusception was consistent with findings from previous studies conducted in similar settings, which utilized both hydrostatic and pneumatic reduction methods.

## INTRODUCTION

Intussusception, the telescoping of one bowel segment into another, primarily affects children under two years of age, with a higher prevalence in boys, and is a leading cause of acute bowel obstruction in infants and toddlers. Approximately 90% of cases are ileocolic in type.^1,2^ Ultrasound (USG) has emerged as the most reliable imaging modality for diagnosing intussusception.^3^ While both surgical and nonsurgical approaches are used globally, image-guided (USG or fluoroscopy-guided) enema reduction is the internationally preferred nonsurgical treatment for uncomplicated cases.^4,5^ The technique of USG-guided hydrostatic reduction using normal saline was first introduced by Kim et al. in 1982.^6^

In Nepal, the use of this therapeutic procedure has been increasing, necessitating an evaluation of its outcomes. This study aims to assess the effectiveness, and outcomes of USG-guided hydrostatic reduction for treating intussusception in children.

## METHODS

This observational cross-section study was conducted at Birat Medical College & Teaching Hospital, which is a tertiary care center at Biratnagar, Morang, Nepal. The study was conducted from February 2023 to July 2024. Ethical approval was taken from the Institutional Review Committee (Reference number: 287/2023). All patients who presented to the emergency and were diagnosed radiologically as intussusceptions were considered as the study population. Patients with symptoms of less than four days and undergoing hydrostatic reduction were included in the study. Patient with signs of peritonitis, abdominal distention, signs of prolapsed intussusceptum from the rectum, and unavailability of consent from a legally authorized representative were excluded. Written informed consent was obtained from legally authorized representative of the children. This procedure was done in all confirmed patients with acute intussusception in our center for therapeutic management of acute intussusceptions as routine procedure.

The variables of study included; age, gender, duration of symptoms, results of treatment and follow up of the patients were noted . The time required to perform USG guided hydrostatic reduction, number of times procedure were done, amount of fluid required during reduction and duration from admission were noted. Successful reduction was achieved when there was complete disappearance of intussusception with free flow of saline from caecum into terminal ileum through ileocaecal valve with distended ileal loops with fluid and absence of intussusceptum on post reduction abdominal ultrasound examination. Failed reduction was said when there is incomplete reduction or perforation of gut.

After proper and adequate resuscitation of the patient, nasogastric tube was inserted. After administration of intravenous medications and all other necessary preparations for surgery was also done if there was failed reduction or there was any complications during the reduction as safeguard. The patient was taken to radiological department for radiological examination. After initial abdominal ultrasound for confirmation of intussusception using high end Voluson S10 ultrasound machine (GE Healthcare, USA) with linear array transducer of 7-12MHz frequency. No sedation was used during the procedure with patient was held in position by radiology staff. The team comprises of radiologist, pediatrician, pediatric surgery, anesthesiologist, nurses and radiology staffs.

The patient was placed in supine position during USG guided hydrostatic reduction with an appropriate Foley catheter (14F - 18F) was inserted into the rectum and balloon inflated with warm normal saline was suspended into drip stand above 100cm from the level of table and was allowed to flow into colon under the gravity.^7^ The retrograde flow of saline and decrease in size of intussusceptum during reduction was observed using ultrasound machine. Once complete reduction was achieved, the fluid was evacuated from the colon by connecting a drainage bag to the rectal tube and allowing the fluid to drain under gravity. The abdomen was then re-examined to determine whether there was any residual lesion or recurrence of intussusception. A total of 3 attempts were allowed, with each reduction lasting 3 to 5 min with an interval of less than 3 min. Even after the third attempts, if the reduction was failed than the procedure were stopped. Surgery was preferred in cases of failed reduction, partially reduced intussusception and in cases of those had complications. After successful USG guided hydrostatic reduction, all patients were kept in the ward for observation for at least 24 hour to evaluate for complications and recurrence with follow up USG on next day.^7^ The patients were followed up with USG after one week also to see for any complications and recurrence.

Data collected was analyzed using Microsoft excel and SPSS Statistics for Windows, version 16.0 (SPSS Inc., Chicago, Ill., USA). Results were expressed in mean ± standard deviation for continuous data, median for non-continuous data and frequencies for categorical data.

## RESULTS

A total of 95 children with 98 intussusceptions were managed during the study period. Out of 95 children, 14 (17.24%) children with 14 intussusceptions were excluded from the study due to presence of signs of peritonitis or abdominal distension or rectal prolapse and they all were primarily managed with surgery. Finally, 81 children with 84 intussusceptions fulfilling the inclusion criteria were included in the study.

Out of 81 children, 60 (74.07%) were male and 21 (25.93%) were female with male to female ratio of 2.86. The age range of the patients was from 3 months to 60 months with a median of 8 months (IQR: 5-11). There were 45 (55.56%) of intussusception occurring between 6-12 months (Table 1).

In this study, 30 (37.04%) patients presented within 24 hours of symptom, 39 (48.15%) patients presented between 24-48 hours and 12 (14.81%) after 48 hours of symptoms. Abdominal pain was seen in all patients 81 (100%) and vomiting in 72 (88.89%) patients (Table 2). History of preceding respiratory or gastrointestinal infection was seen in 49 patients (60.49%).

**Table 1:** Age distribution of patient with intussusception undergoing ultrasound guided hydrostatic reduction (n=81).

Age Group	n (%)
<6 months	22 (27.16)
6-12 months	45 (55.56)
13-18 months	7 (8.64)
19-24 months	4 (4.94)
>24 months	3 (3.7)

**Table 2:** Presenting complains of the patients with intussusception undergoing ultrasound guided hydrostatic reduction (n=81).

Complains	n (%)
Abdominal pain	81 (100)
Vomiting	72 (88.89)
Passage of red current jelly stool	65 (80.25)
Abdominal mass	73 (90.12)
Fever	40 (49.38)
Dehydration	37 (45.67)

**Table 3:** Ultrasound findings of the patients (n-81) with intussusceptions undergoing ultrasound guided hydrostatic reduction (n=84).

Ultrasound findings	n (%)
Intra-abdominal mass	84 (100)
Target sign	84 (100)
Pseudo-kidney sign	84 (100)
Dilated fluid filled bowel	46 (54.76)
Mesenteric lymphadenopathy	17 (20.23)
Ascites	15 (17.86)

In all intussusceptions i.e 84 (100%) in 81 patients, intraabdominal mass with pseudo-kidney sign and atypical target appearance was seen during ultrasonographic evaluation (Table 3).

Out of 84 intussusception, Ileocolic intussusception was seen in 74 (88.09%) cases, colocolic in 8 (9.53%) cases and ileoileal type in 2 (2.38%) cases. The most distal end of the intussusception was at the descending colon seen in 17 (20.24%) patients, at the ascending colon seen in 21 (25%) patients and at the transverse colon seen in 46 (54.76%) patients. USG guided hydrostatic reduction was successful in 78 (92.85%) cases in our study. In our study, hydrostatic reduction was not successful in 6 (7.15%) cases which were managed by surgery. There were 3 (3.57%) patients who had recurrence and repeat successful hydrostatic reduction was done in all of them. One patient had recurrence within 24 hours of reduction and two patients had recurrence after 24 hours of reduction during the hospital stay. The duration of procedure for successful reduction was ranged from three minutes to twenty five minutes (median-6 minutes) with majority of them (55 out of 78) showing successful reduction between five to ten minutes from the time when normal saline was released (mean-6.60±3.63 minutes).

## DISCUSSION

This study's aim was to evaluate the outcomes and effectiveness of USG-guided hydrostatic reduction of acute uncomplicated intussusceptions. Successful reduction of USG-guided hydrostatic reduction was seen in 78 (92.85%) of the patients. The rate of successful USG-guided hydrostatic reduction of intussusception in our study was 92.85% and similarly high rate of successful reduction was seen in the majority of the studies. ^3,5 8-12^ A study by Rajkarnikar R et al^12^ from Nepal showed a 92.1% success rate of USG-guided hydrostatic reduction of intussusception in their study which is similar to the success rate of hydrostatic reduction in our study. Another two studies from Nepal on pneumatic reduction of intussusception showed a success rate of 92% and 96% respectively which is also similar to the success rate of hydrostatic reduction in our study.^14,15^ USG-guided hydrostatic reduction is preferred over fluoroscopy-guided pneumatic reduction as there is a lack of radiation exposure, easily available, more economical, and more chance of success. There is better visualization of intussusception and more chance of reduction due to the use of saline as an enema in the case of USG-guided hydrostatic reduction in comparison to USG-guided pneumatic reduction as air gives posterior dirty shadowing leading to difficulty in image visualization. The majority 55 (73.33%) of the successful hydrostatic reduction in our study were reduced within five to ten minutes from the time when normal saline was released and the duration of successful reduction ranged from three minutes to twenty-five minutes making it shorter procedure than surgery which normally not takes less than forty minutes. This difference in time and ease of procedures provided positive findings in favor of ultrasound-guided hydrostatic reduction in the initial management of intussusception. Also, there is a lack of radiation during the process and it is an easily available, safe, and simple procedure associated with less morbidity and mortality with a shorter duration of hospital stay and causes less financial burden to the health care system.

Surgery is reserved for patients with failed reduction, partial reduction, or patients with signs of peritonitis and bowel obstruction, or patients with long-duration of symptoms. In our study, 6 (7.14%) patients were managed by surgery due to failed hydrostatic reduction. Five (83.33%) patients were managed by open reduction while resection and anastomosis were done in one (17.74%) patient.

Intussusception was more commonly seen in male patients than in female patients with a male-to-female ratio of 2.86 in our study with similar male predominance seen in many studies .^3,5, 8-12^ The most common age group involved in our study was between 6 months to 12 months which was similar to many studies^6,9-11^ however it varied from many other studies.^3, 8 12^ The exact reason for this variation was not well known but can be due to the different geographical location, dietary patterns and age of weaning of the infants may be possible reasons. Most of the patients in our study presented to the hospital within 24-48 hours of symptoms similar to other studies.^8,10-11^ Late presentation was more commonly seen in developing countries due to lack of knowledge, ignorance, poverty, and not easily available proper health services. The most common presenting symptom seen in our study was abdominal pain similar to other studies^8,10-11^ while abdominal mass, intermittent cry, and vomiting were the most common presenting symptoms in other studies.^12,13^

The presentation and symptoms of intussusception are not fixed and it is nonspecific. It can be seen in children with a variety of symptoms. The difference in presenting symptoms in different studies was due to differences in the time of presentation to the hospital and also due to different stages of the disease. Most of the patients presented with complaints of abdominal pain and intermittent crying. Due to preceding viral respiratory or gastrointestinal infection, there is hyperplasia of Peyer's patch of small bowel which acts as lead point for intussusception. There is more chance of intussusception in children preceding viral infections. There is a high chance of detection of the virus in the stool of patients with intussusception.^11^ In our study, the Ileocolic type of intussusception was most commonly seen in 88.09% of the patients which was similar to the findings of most of the studies and the cause is idiopathic. ^3,5, 8-12^ The lesser common types seen in our study were the ileoileal and colocolic types. In the case of pathological lead points like focal mass lesions, meckel's diverticulum, duplication cyst, polyp, or focal bowel wall thickening, any part of bowel loops can be involved in intussusception.

Transverse colon was the common site for the most distal end of intussusception in our study and was seen in 54.76% of patients which was similar to findings seen in the majority of the studies.^3,11^ Due to the variation in the length of the mesentery in different children, time and duration of the presentation of the patients to the hospital, the sites of the intussusception may vary and some patients coming with prolapsing intussusception through the anus in very late presenting and neglected cases as intussusceptions move from right to left side with time.^11^

This was a single institutional study done in one region of the country with a shorter duration of study and a small sample size which limits the generalizability of the findings. A larger number of cases would have availed better analysis and basis for critical comparison with other published larger series.

## CONCLUSIONS

The success rate of ultrasound-guided hydrostatic reduction (USG-HR) using normal saline for uncomplicated intussusception was found to be consistent with outcomes reported in previous studies conducted in comparable settings. These studies employed both hydrostatic and pneumatic reduction methods, demonstrating similar efficacy in achieving successful reduction. The high success rate observed in this study aligns with established evidence supporting USG-HR as a reliable and effective nonsurgical treatment for pediatric intussusception.
